# *FAMLF* is a target of miR-181b in Burkitt lymphoma

**DOI:** 10.1590/1414-431X20175661

**Published:** 2017-05-04

**Authors:** J.G. Li, Y. Ding, Y.M. Huang, W.L. Chen, L.L. Pan, Y. Li, X.L. Chen, Y. Chen, S.Y. Wang, X.N. Wu

**Affiliations:** 1 Department of Hematology, Fujian Institute of Hematology, Fujian Medical University Union Hospital, Fuzhou, China; 2 Union Clinical Medical College, Fujian Medical University, Fuzhou, China; 3 School of Public Health, Fujian Medical University, Fuzhou, China

**Keywords:** Burkitt lymphoma, FAMLF, miR-181b, Gene expression, RQ-PCR

## Abstract

Burkitt lymphoma (BL) is a highly malignant non-Hodgkin's lymphoma that is closely related to the abnormal expression of genes. Familial acute myelogenous leukemia related factor (*FAMLF*; GenBank accession No. EF413001.1) is a novel gene that was cloned by our research group, and miR-181b is located in the intron of the *FAMLF* gene. To verify the role of miR-181b and *FAMLF* in BL, RNAhybrid software was used to predict target site of miR-181b on *FAMLF* and real-time quantitative PCR (RQ-PCR) was used to detect expression of miR-181b and *FAMLF* in BL patients, Raji cells and unaffected individuals. miR-181b was then transfected into Raji and CA46 cell lines and *FAMLF* expression was examined by RQ-PCR and western blotting. Further, Raji cells viability and proliferation were detected by MTT and clone formation, and Raji cell cycle and apoptosis were detected by flow cytometry. The results showed that miR-181b can bind to bases 21–42 of the *FAMLF* 5′ untranslated region (UTR), *FAMLF* was highly expressed and miR-181b was lowly expressed in BL patients compared with unaffected individuals. *FAMLF* expression was significantly and inversely correlated to miR-181b expression, and miR-181b negatively regulated *FAMLF* at posttranscriptional and translational levels. A dual-luciferase reporter gene assay identified that the 5′ UTR of *FAMLF* mRNA contained putative binding sites for miR-181b. Down-regulation of *FAMLF* by miR-181b arrested cell cycle, inhibited cell viability and proliferation in a BL cell line model. Our findings explain a new mechanism of BL pathogenesis and may also have implications in the therapy of FAMLF-overexpressing BL.

## Introduction

Burkitt lymphoma (BL) is a highly aggressive non-Hodgkin's B-cell lymphoma and affects children and adolescents more commonly. Patients often present with a large tumor, a high tumor burden and mortality due to the short doubling time of the tumor (1). Studies have shown that Epstein-Barr virus (EBV) infection and eight chromosome *MYC* oncogene translocations were involved in the pathogenesis of BL. Activation of the *MYC* gene may promote cell proliferation and malignant transformation, and lead to the occurrence of tumors (2). However, EBV infection and *MYC* oncogene translocation were not detected in some BL cases, indicating that the complete molecular mechanisms of the pathogenesis of BL have not been fully elucidated.

Familial acute myelogenous leukemia related factor (*FAMLF*; GenBank accession No. EF413001.1) is a novel leukemia-associated gene that was cloned and identified by a series of molecular biology techniques from a large family with a high incidence of leukemia in Fujian, China. The *FAMLF* gene is located on human chromosome 1q32.1; its full-length cDNA is 2313 bp and encodes an 82-amino acid polypeptide (protein accession no. ABN58747) (3–5). Studies have shown that *FAMLF* was highly expressed in acute myeloid leukemia and BL patients, NB4 acute promyelocytic leukemia cells, U937 macrophage-like cells, K562 myeloid leukemia cells, U266 myeloma cells, HL60 promyelocytic cells, CA46 lymphoma cells, and especially in Raji BL cells, while low expression was observed in unaffected individuals. *FAMLF* may be involved in hematopoietic neoplasms by promoting cell proliferation and preventing cell differentiation (6,7).

Micro RNAa (miRNAs) are small non-coding RNA molecules that contain approximately 19-24 nucleotides that down-regulate gene expression, primarily by base-pairing to the 3′ untranslated region (UTR) of target mRNAs (8). A previous study showed that miRNAs are widely involved in many pathophysiological processes and are associated with a variety of malignant tumors (9). Multiple miRNA expression and regulation abnormalities were also found in BL (10–13), indicating that miRNAs are closely associated with the pathogenesis of BL.

miR-181b is located in the intron of the *FAMLF* gene, making *FAMLF* the host gene of miR-181b. Previous studies have shown that intronic miRNAs and host genes are closely related and that intronic miRNAs could negatively regulate expression of host genes (14–17). The aim of this study was to evaluate the expressions of miR-181b and *FAMLF* in BL and in Raji BL cells.

## Material and Methods

### Patient samples

The study was approved by Fujian Medical University Ethics Committee. Forty-five samples were obtained with written informed consent from 30 patients diagnosed with BL at Fujian Institute of Hematology and from 5 unaffected individuals. Samples were obtained also from 2 BL cell lines. Of the 30 patients, 19 were male and 11 were female, the median age was 13 years (range 1–42 years), 6 of the 45 samples were from patients who were in remission, and 2 were from patients with recurrent disease. Clinical characteristics of the cohort are listed in Table 1. Expressions of miR-181b and *FAMLF* were detected in a paired manner in each specimen.

**Table 1:**
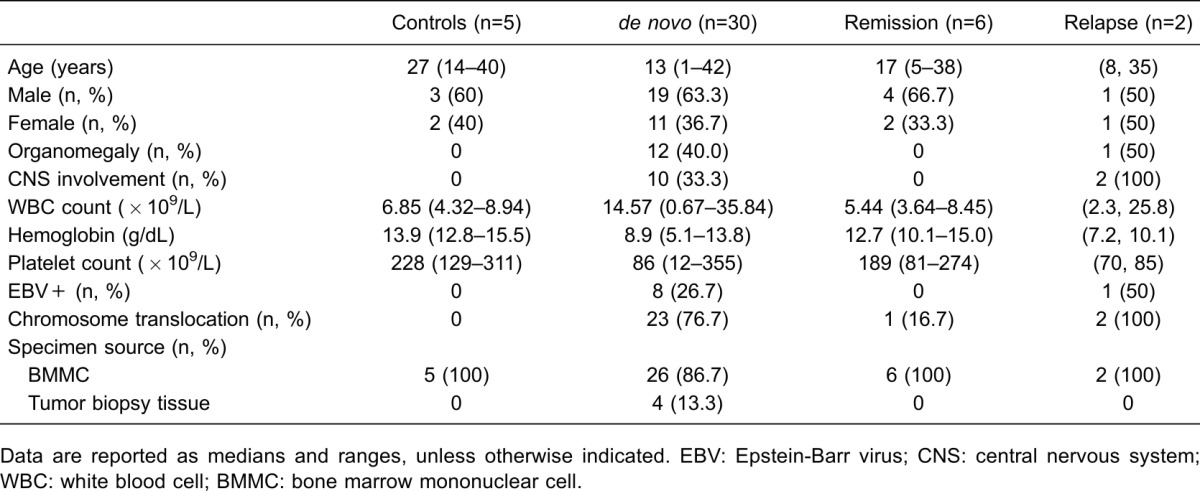
Clinical characteristics of unaffected individuals (controls) and Burkitt lymphoma (BL) patients.

### Real-time quantitative PCR (RQ-PCR) for *FAMLF*

Total RNA was extracted from bone marrow mononuclear cells or ground tumor tissue by using TRIzol Reagent (Invitrogen, USA), first-strand cDNA was synthesized using a RevertAid TM First Strand cDNA Synthesis Kit (Fermentas, Canada), and the following specific primers were used to amplify *FAMLF* by RQ-PCR: forward primer, 5′-ACCGTTTTGAAATTAGATCC-3′; reverse primer, 5′-CGATTGAACTAAAAGA AATGAC-3′. β-actin was used as the internal control gene: forward primer, 5′-AGTGTGACGTGGACATCCGCAAAG-3′; reverse primer, 5′-ATCCACATCTGC TGGAAGGTGGAC-3′. All primers were synthesized by Shanghai Invitrogen Biotechnology Co. Ltd. (China). RQ-PCR was performed on a 7500-thermal cycler (ABI, USA) using FastStart Universal SYBR Green Master Mix (Roche, USA) with the following program: 50°C for 2 min and 95°C for 10 min, followed by 40 cycles of 95°C for 15 s and 58°C for 1 min. All samples were run in triplicate, and the 2^-ΔCt^ (^Δ^Ct=CT*FAMLF*-CT*β-actin*) method was used to estimate the relative expression level of the *FAMLF* gene.

### RQ-PCR for miR-181b

MicroRNA was extracted from bone marrow mononuclear cells or ground tumor tissue by using a miRNeasy Mini Kit (QIAGEN, Germany). U6 was used as the internal control for miRNA and reverse transcription-specific primers with stem loop structures were designed according to Chen et al. (18): miR-181b, 5′-GTCGTATCCAGTGCAGGGTCCGAGGTATTCGCACTGGATACGACAACCCACCG-3′; U6, 5′-GTCGTATCCAGTGCAGGGTCCGAGGTATTCGCACTGGATACGACAAAAATATG-3′. Reverse transcription was performed on a 2720 thermal cycler (ABI) using a TaqMan microRNA Reverse Transcription Kit (ABI). The following specific primers were used to amplify miR-181b and U6: miR-181b forward primer, 5′-AACATTCATTGCTGTCGGTGGGT-3′; U6 forward primer, 5′-GCGCGTCGTGAAGCGTTC-3′; universal reverse primer, 5′-GTGCAGGGTCCGAGGT-3′. RQ-PCR was performed on a 7500-thermal cycler (ABI) using the TaqMan microRNA Assay (ABI) with the following program: 50°C for 2 min and 95°C for 10 min, followed by 40 cycles of 95°C for 15 s and 60°C for 1 min. All samples were run in triplicate, and the 2^-ΔCt^ (ΔCt=CT_miR-181b_-CT_U6_) method was used to estimate the relative expression level of miR-181b.

### miR-181b transfection and *FAMLF* assays

The Raji and CA46 cell lines were purchased from the cell library of the Chinese Academy of Medical Sciences. miR-181b mimics and miR-181b negative controls (NC) were synthesized by Guangzhou Ribobio Co. Ltd. (China). After recovery, cells were cultured in RPMI-1640 medium containing 10% fetal bovine serum (Biosera, France) at 37°C and 5% CO_2_ with maximum humidity. The experiments were divided into three groups: the blank control group (group Raji and group CA46), the negative control group transfected with miR-181b NC (group Raji/NC and group CA46/NC) and the experimental group transfected with miR-181b mimics (group Raji/miR-181b and group CA46/miR-181b). Two independent experiments were performed and three biological replicates were performed for each experiment.

Raji and CA46 cells were seeded in 24-well plates. miR-181b mimics (30 pmol) or miR-181b NC (30 pmol) in 50 µL of medium were mixed with 2 µL of Lipofectamine 2000 (Invitrogen, USA) transfection reagent dissolved in 50 µL of the same medium and allowed to stand at room temperature for 20 min. The resulting 100-µL transfection solutions were then added to each well containing 400 µL of medium. After being incubated for 5 h, miRNA was extracted and RQ-PCR was performed to detect the miR-181b transfection efficiency as described above.

At 24 h after transfection, total RNA was extracted, and RQ-PCR was performed to detect the expression of *FAMLF* at the mRNA level as described above. Total protein was extracted using a ProteoPrep Total Extraction Sample Kit (Sigma, USA), and the level of *FAMLF* protein was confirmed with a monoclonal anti-*FAMLF* antibody (Clone No. 269.3, Abmart, USA) according to standard procedures for western blotting. Normalization was performed using a monoclonal anti-actin antibody (Clone No. AC-16, Abmart), and the band intensity was quantified with the Doc Gel 2000 imaging analysis system (Bio-Rad, USA).

### Dual-luciferase reporter gene assays

Wild-type and mutated 5′ UTRs of the *FAMLF* gene were, respectively, amplified by using the following primers incorporating the SacI and Xbal restriction sites: *FAMLF* 5′ UTR forward primer, 5′-GAGCTCAGAACTGCAGATAGTACAGC-3′; *FAMLF* 5′ UTR reverse primer, 5′-TCTAGATAAAAAATGGACTAGTGGACTG G-3′; mutated *FAMLF* 5′ UTR forward primer, 5′-GAGCTCTAGACCATCACCATC GACTGTCTGAGCACA-3′; mutated *FAMLF* 5′ UTR reverse primer, 5′-TCTAGATA AAAAATGGACTAGTGGACTGG-3′. The wild type and mutated *FAMLF* 5′ UTR sequences were individually cloned upstream of the Renilla luciferase (*hRluc*) gene in the dual-luciferase reporter gene vector pmiR-RB-Report™ (Ribobio, China) to construct the wild-type recombinant plasmid vector pmiR-RB-Report™/*FAMLF*-5′ UTR-WT (*FAMLF*-WT) and mutated recombinant plasmid vector pmiR-RB- Report™/*FAMLF*-5′ UTR-MT (*FAMLF*-MT). Wild-type and mutated inserts were confirmed by sequencing. The 293T cells were co-transfected in 24-well plates with 2 µg recombinant plasmid vectors and 30 pmol miR-181b using 2-µL lipofectamine 2000 (Invitrogen, USA) according to the manufacturer's protocol. After a 48-h incubation, *hRluc* activity was detected by chemiluminescence and using the Dual-Luciferase Reporter Assay System (Promega, USA). Normalization was performed using the Firefly luciferase (*hluc*) gene. Two independent experiments were performed and three biological replicates were performed for each experiment.

### Cell viability and cell cycle assays

After being transfected with miR-181b mimics or miR-181b NC, cells were collected from culture and seeded in 96-well assay plates with three replicates. After being incubated for 0, 24, 48 and 72 h, 3-(4, 5-dimethylthiazol-2-yl)-2, 5- diphenyltetrazolium bromide (MTT) (Sigma, USA) was added, and the cells were incubated at 37°C for 4 h followed by the addition of dimethyl sulfoxide (Sigma) to dissolve the formazan crystals. Absorbance was read at 570 nm. Two independent experiments were performed.

For cell cycle analysis, the cells were seeded on 96-well assay plates with three replicates. The transfected Raji cells were incubated for 0, 24 and 48 h, followed by the addition of propidium iodide (Sigma), and the cells were incubated at 4°C for 30 min. The DNA content was detected by FC-50 flow cytometry (Beckman Coulter, USA) and cell cycle analysis was performed. Two independent experiments were performed.

### Cell proliferation and cell apoptosis assays

The effects of miR-181b on the proliferation of Raji cells was analyzed by the clonogenic assay. Briefly, cells were collected from culture and seeded in 10-cm culture dishes at density of 10^4^ cells per dish with 6 replicates. Cells were incubated for 1 week at 37°C with 5% CO_2_ until the cells in control dishes have formed visual colonies that were of a substantially good size. At the end of incubation period, the numbers of cell colonies (greater than 50 cells) were counted by light microscopy.

Apoptosis assays were performed according to the introduction in Annexin V-FITC Apoptosis Detection Kit (Sigma) as follows: after the transfected Raji cells were incubated for 0, 24, 48 and 72 h, the cells were harvested and washed with pre-chilled phosphate buffered saline (PBS) twice. A total of 10^6^ Raji cells were stained with FITC-labeled Annexin V and propidium iodide. The stained cells were analyzed by FC-50 flow cytometry (Beckman Coulter) for apoptosis assay.

### Statistical analysis

The statistical software SPSS19.0 (USA) was used to draw a scatter diagram of the expression of miR-181b and *FAMLF* and to analyze their correlation. For cell viability, cell cycle, cell proliferation and cell apoptosis assays, data are reported as means±SD. Analysis of variance (ANOVA) and group q-tests were performed and P<0.05 was considered to be statistically significant.

## Results 

### miR-181b expression was significantly inversely correlated to *FAMLF* expression in BL patients

**Figure 1:**
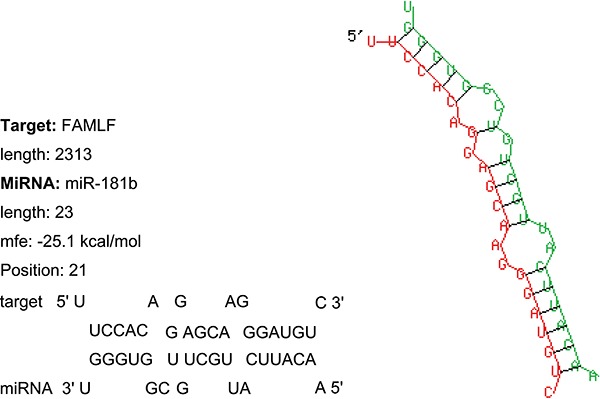
miR-181b target site prediction for *FAMLF* by the RNAhybrid program. miR-181b bound with bases 21-42 of the *FAMLF* 5′ untranslated region in an incomplete complementary manner.

**Figure 2:**
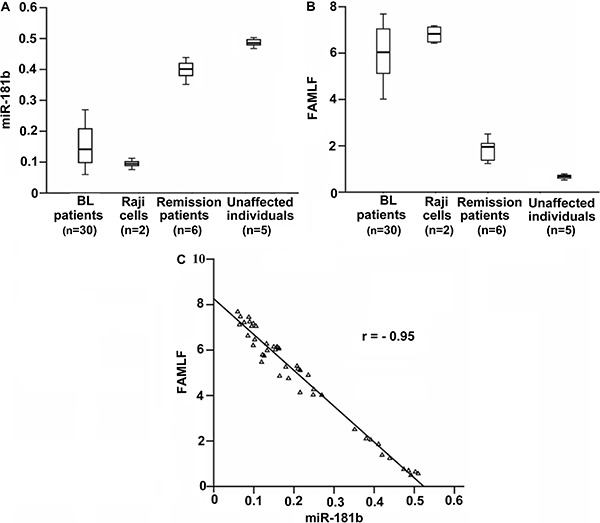
Expression of miR-181b and *FAMLF* in Burkitt lymphoma (BL) patients, Raji BL cells, remission patients and unaffected individuals. *A*, miR-181b was little expressed in BL patients and Raji BL cells, but highly expressed in remission patients and unaffected individuals. *B*, *FAMLF* was highly expressed in BL patients and Raji BL cells, but little expressed in remission patients and unaffected individuals. *C*, *FAMLF* expression was inversely correlated with miR-181b expression. Data are reported as means±SD. P<0.01 (Pearson test).

miR-181b target site prediction for *FAMLF* was performed using the RNAhybrid program (http://bibiserv.techfak.uni-bielefeld.de/rnahybrid/) (19). We found that miR-181b can bind to bases 21–42 of the *FAMLF* 5′ UTR in an incomplete complementary manner (Figure 1). To evaluate this putative interaction, we first detected the expression levels of miR-181b and *FAMLF* and examined the correlation of the expression in BL patients, Raji BL cells and unaffected individuals. We found that miR-181b was little expressed and *FAMLF* was highly expressed in BL patients and Raji BL cells, but miR-181b was highly expressed and *FAMLF* was little expressed in remission patients and unaffected individuals (Figure 2A and B). The expression of *FAMLF* was significantly inversely correlated to the expression of miR-181b, with the coefficient of correlation in the full set of 45 samples being -0.95 with P<0.01 (Figure 2C).

### miR-181b down-regulated the expression of the *FAMLF* gene at posttranscriptional and translational levels

**Figure 3:**
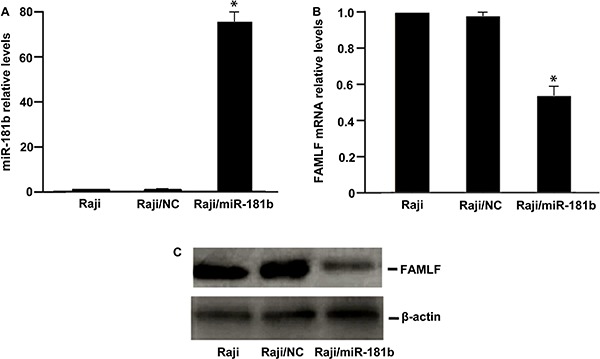
Detection of miR-181b transfection efficiency and *FAMLF* expression in Raji cells. *A*, miR-181b expression was increased 75 times after transfection. *B*, *FAMLF* mRNA expression was reduced by half after transfection. *C*, *FAMLF* protein was reduced by approximately 20% after transfection. Raji group was used as the control (NC) and was normalized. Data are reported as means±SD (n=6). *P<0.01, Raji/miR-181b compared to Raji and Raji/NC (ANOVA).

**Figure 4:**
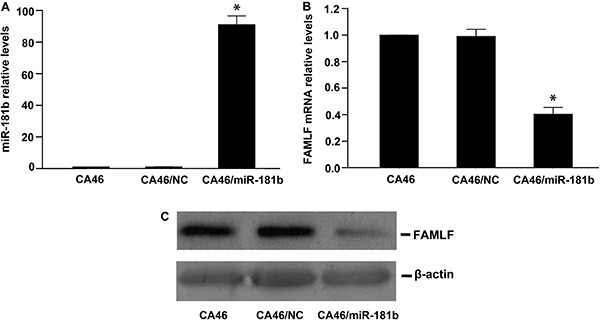
Detection of miR-181b transfection efficiency and *FAMLF* expression in CA46 cells. *A*, miR-181b expression was increased 91 times after transfection. *B*, *FAMLF* mRNA expression was reduced by 40% after transfection. *C*, FAMLF protein expression was reduced by 14% after transfection. CA46 group was used as a control (NC) and was normalized. Data are reported as means±SD (n=6). *P<0.01, CA46/miR-181b compared to CA46 and CA46/NC (ANOVA).

To investigate the effects of miR-181b on *FAMLF* expression, we selected Raji and CA46 BL cells, which express high levels of *FAMLF* and low levels of miR-181b. Synthesized miR-181b mimics were respectively transfected into Raji and CA46 cells and transfection efficiency was detected. We found that miR-181b expression was significantly increased in Raji cells (Figure 3A) and CA46 cells (Figure 4A) after transfection. Next, the expression of *FAMLF* was detected at both the mRNA and protein levels. We found that *FAMLF* mRNA was reduced in Raji cells (Figure 3B) and CA46 cells (Figure 4B) transfected with miR-181b, and that *FAMLF* protein was reduced more compared with *FAMLF* mRNA (Figures 3C and 4C). These results showed that miR-181b can down-regulate the expression of *FAMLF* gene at both the mRNA and protein levels.

### miR-181b directly interacted with the 5′ UTR of *FAMLF* by incomplete complementary base pairing

Unlike regulation by complete complementary base pairing in plants, miRNAs in mammals regulate the expression of target mRNAs mainly by incomplete complementary base pairing. In a dual-luciferase assay, the existence of an interaction between miR-181b and wild-type *FAMLF* mRNA should reduce *hRluc* gene activity, while mutated *FAMLF* mRNA would not bind with miR-181b and therefore would not alter the activity of the reporter gene. The data presented in Figure 5 shows a significant suppression of *hRluc* gene activity in the *FAMLF*-WT/miR-181b group, but no changes in the control groups. This result indicated a direct effect of miR-181b on *FAMLF*.

**Figure:**
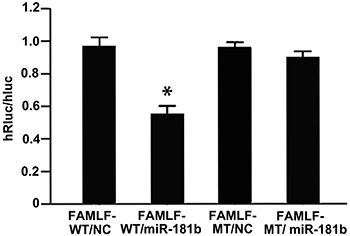
Dual-luciferase reporter gene assay. *Renilla luciferase* gene activity was reduced by half in 293T cells transfected with miR-181b mimics and the wild-type *FAMLF* 5′ UTR sequence (FAMLF-WT/miR-181b), while miR-181b negative controls (FAMLF-MT/NC) and the mutated *FAMLF* 5′ UTR sequence (FMALF-MT/miR-181b) caused no statistical changes in luciferase gene expression. Data are reported as means±SD (n=6). *P<0.01, compared to *FAMLF*-WT/NC, *FAMLF*-MT/NC and *FAMLF*-MT/miR-181b (ANOVA). *hRluc*: Renilla luciferase reporter gene; *hluc*: firefly gene.

### *FAMLF* down-regulation by miR-181b inhibited cell viability and arrested cell cycle in Raji cells

**Figure 6:**
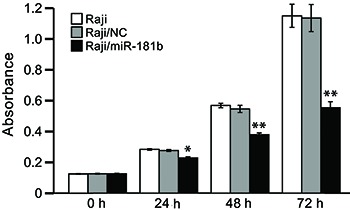
Effect of miR-181b on Raji cell viability. After transfection for 24, 48, and 72 h, cell viability was significantly decreased in the Raji/miR-181b group compared with the Raji and Raji/normalized control (NC) groups, *P<0.05, **P<0.01 (ANOVA). Data are reported as means±SD (n=6).

**Figure 7:**
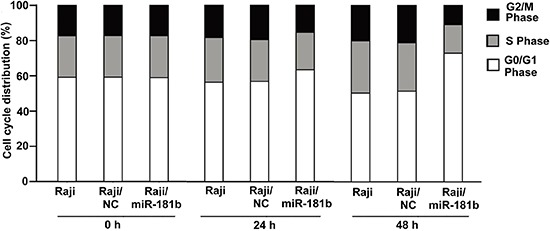
Flow cytometry analysis showing the Raji cell cycle distribution after 24 and 48 h of transfection. The percentage of cells was increased in G0/G1 phase (58.98-72.94%) and was reduced in G2/M and S phase after transfection of miR-181b. P<0.05, Raji/miR-181b compared to Raji and Raji/NC (ANOVA). NC: normalized control. Data are reported as means (n=6).

The previous results have shown that miR-181b directly down-regulated the expression of *FAMLF* in Raji cells. We next studied the effect of *FAMLF* down-regulation by miR-181b on cell viability. MTT assays showed that cell viability was significantly decreased in the Raji/miR-181b group compared with control groups, with P<0.01 (Figure 6). In addition, we also analyzed the cell cycle distributions after transfection for 0, 24 and 48 h and found that the relative amount of cells in G0/G1 phase was increased (58.98–72.94%) in the Raji/miR-181b group, whereas the percentage of cells in S and G2/M phase was reduced, with P<0.05. The cell cycle distribution in the Raji/NC group had no obvious changes compared with the Raji group (Figure 7). These results showed that miR-181b inhibited Raji cells viability and arrested cell cycle by *FAMLF* down-regulation.

### *FAMLF* down-regulation by miR-181b inhibited cell proliferation but had no effect on apoptosis in Raji cells

**Figure 8:**
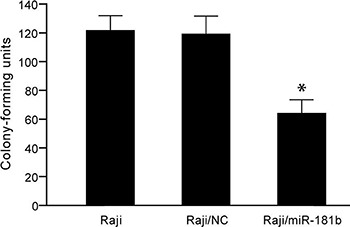
*FAMLF* down-regulation by miR-181b significantly reduced the colony-formation ability of Raji cells. Colony-forming units were decreased from 119 to 64 after transfection of miR-181b. *P<0.01, Raji/miR-181b compared to Raji and Raji/NC (ANOVA). Data are reported as means±SD (n=6).

**Figure 9:**
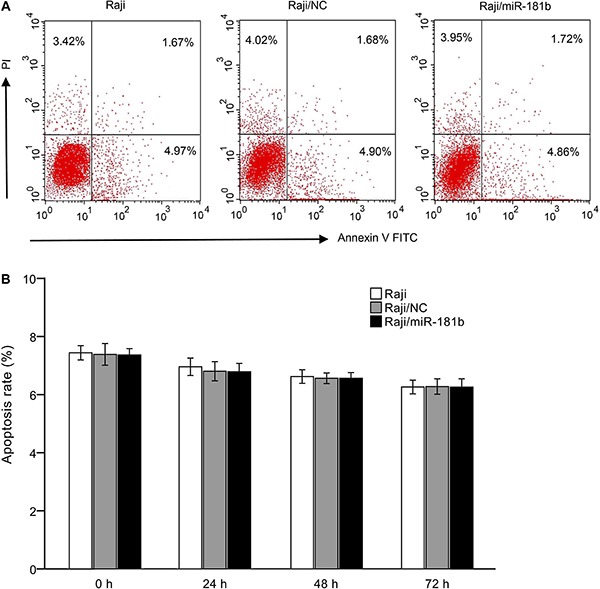
Effect of miR-181b transfection on Raji cell apoptosis. *A*, Flow cytometry apoptosis scatter diagram after transfection for 48 h. *B*, After transfection for 0, 24, 48, and 72 h, total apoptosis rates (early apoptosis and late apoptosis) had no obvious changes in Raji/miR-181b group compared with control groups (Raji and Raji/NC) (P>0.05, ANOVA). Data are reported as means±SD (n=6).

The clonogenic assay is a commonly used way to evaluate cell proliferation ability. In this experiment, we observed a very strong decrease in colony-forming units in Raji/miR-181b group compared with control groups, with P<0.01 (Figure 8). The results showed that *FAMLF* down-regulation by miR-181b inhibited cell proliferation in Raji cells. However, we found no significant change in apoptosis rate in Raji/miR-181b group compared with control groups, with P>0.05 (Figure 9).

## Discussion

miR-181b is a member of the miR-181 family that is located on human chromosome 1q32. Recent studies have shown that miR-181b, as a small RNA molecule, was involved in cell differentiation and development as well as proliferation and apoptosis through the down-regulation of target genes (20,21). miR-181b plays a role in cancer enhancement by down-regulating anti-oncogenes. Tong et al. (22) reported that miR-181b promoted prostate cancer cell proliferation by regulating DAX-1 expression. However, in most instances, miR-181b plays a role of cancer suppressor by down-regulating oncogenes. Wang et al. (23) reported that miR-181b inhibited glioma cell proliferation by targeting cyclin B1. Pekarsky et al. (24) reported that the expression levels of Tcl1 are inversely correlated with miR-181 expression and that Tcl1 expression is regulated by miR-181 in chronic lymphocytic leukemia, but the role of miR-181b has not been described in BL. Our research showed that miR-181b was down-regulated and might act as a cancer suppressor in BL.

The *FAMLF* gene is a novel gene cloned from leukemia patients that is not only highly expressed in leukemia but also in other tumors, especially in BL, indicating its widespread cancer promoting activity. However, the mechanism by which *FAMLF* gene is highly expressed and how is it regulated in tumors is still unclear. Recent studies have indicated that more than 30% of the protein coding genes may be the target genes of miRNAs (25). About half of the known miRNA genes are located within introns of host genes and an intronic miRNA might regulate the expression of the host gene (26–28). As *FAMLF* is the host gene of intronic miR-181b, we postulated that miR-181b regulated the expression of *FAMLF*. To verify this assumption, RQ-PCR was first performed to detect expression levels of *FAMLF* and miR-181b in BL patients with different clinical characteristics. We found that the *FAMLF* gene was up-regulated in the BL patients with a low expression of miR-181b and down-regulated in the BL patients with a high expression of miR-181b. *FAMLF* expression was therefore significantly and inversely correlated to miR-181b expression, indicating a possible negative regulation between intronic miR-181b and the host gene *FAMLF*. We also found that expression of *FAMLF* was high in *de novo* BL patients and low in the patients in remission, and it was higher in patients with extensive spread than patients with localized lesions, and related to tumor burden. These results showed that the abnormal expression of miR-181b and *FAMLF* was related to the pathogenesis of BL.

miRNA mimics are synthetic miRNAs that can simulate the high expression of endogenous mature miRNA in cells and are simple and efficient tools for studying the regulation of target genes (29). To further verify the role of miR-181b in the regulation of *FAMLF*, we transfected miR-181b mimics into the Raji cell line and then detected the expression of *FAMLF*. The results showed that after transfection of miR-181b mimics, miR-181b expression was increased significantly in the Raji cell line and *FAMLF* mRNA expression level was decreased by half, while the protein expression level was reduced to 1/5 of control levels. Although some earlier studies reported that miRNA only inhibited expression of target genes at translation level and did not affect the abundance of mRNA in mammals, many subsequent research results indicated that miRNAs can not only inhibit translation of the target mRNA, but also directly induce its degradation by two completely independent mechanisms (30–32). In our experiments, miR-181b down-regulated expression of *FAMLF* at both the mRNA and translation levels.

Down-regulation of *FAMLF* by miR-181b could be explained by a direct interaction (miR-181b::*FAMLF* complementarity) or by an indirect effect. It can be argued that miR-181b interacted with other unknown targets that, in turn, down-regulated the expression of *FAMLF*. To solve this issue, wild-type and binding site mutation sequences of *FAMLF* were individually fused into the 5′ UTR of a luciferase reporter gene and were individually cotransfected into 293T cells with miR-181b mimics, and luciferase reporter gene activity was then detected. We found that *hRluc* gene activity was significantly reduced in the *FAMLF*-WT/miR-181b group, but no changes were seen in the control groups. This result indicated a direct effect of miR-181b on *FAMLF*. Although the traditional mechanism through which miRNAs regulate expression of target genes involves binding to the 3′ UTR of mRNA, increasing number of studies have found that the 5′ UTR and CDS of target genes also contain miRNA binding sites (33,34). miR-181b binding to the 5′ UTR of *FAMLF* mRNA may inhibit the formation of the mRNA 5′ cap structure to affect the stability of mRNA or may prevent the binding of mRNA with the ribosome to inhibit its translation, thus resulting in the down-regulation of *FAMLF* expression.

A proto-oncogene is a normal gene that helps to regulate cell growth and differentiation and could become an oncogene due to mutation or increased expression (35). Based on our study results in BL, we speculate that *FAMLF* may play a role similar to that of a proto-oncogene. In this potential mechanism, low expression of mir-181b leads to dysregulation of *FAMLF*, and overexpression of *FAMLF* may up-regulate expression of genes related to cell proliferation, such as *c-MYC*, through transcription factor activity, resulting in uncontrolled cell proliferation, which may be an important mechanism of the pathogenesis of BL. In Raji cells transfected with miR-181b, we observed the reduction of cells in S and G2/M phase and inhibition of cell viability and proliferation, demonstrating that the down-regulation of *FAMLF* by miR-181b is sufficient to inhibit cell viability and proliferation. These results indicate that miR-181b may be used for therapy in BL overexpressing *FAMLF*.

In conclusion, our study showed that *FAMLF* expression was inversely correlated to miR-181b expression in BL and that miR-181b directly down-regulated the expression of *FAMLF* and inhibited cell viability by binding to the 5′ UTR of *FAMLF*. Our findings explain a new mechanism of the pathogenesis of BL, and miR-181b may be a candidate for therapeutic agents in BL overexpressing *FAMLF*.
